# Neodymium(III) molybdenum(VI) borate, NdBO_2_MoO_4_

**DOI:** 10.1107/S1600536811017806

**Published:** 2011-05-20

**Authors:** Peter Held, Benjamin van der Wolf, Ladislav Bohatý, Petra Becker

**Affiliations:** a Institut für Kristallographie, Universität zu Köln, Greinstrasse 6, D-50939 Köln, Germany

## Abstract

Single crystals of NdBO_2_MoO_4_ were obtained from a molybdenum oxide–boron oxide flux under an air atmosphere. The structure features double chains of edge- and face-sharing distorted [NdO_10_] bicapped square-anti­prisms, which are linked by rows of isolated [MoO_4_] tetra­hedra and by zigzag chains of corner-sharing [BO_3_] groups, all of them running along the *b* axis. The chains of [NdO_10_], chains of [BO_3_] and rows of [MoO_4_] groups are arranged in layers parallel to the *bc* plane.

## Related literature

A rough investigation of subsolidus phase relations in the pseudo-ternary system Nd_2_O_3_ – B_2_O_3_ – MoO_3_ has been reported by Lysanova *et al.* (1983 ▶) and Dzhurinskii & Lysanova (1998 ▶). X-ray powder diffraction data of *Ln*BO_2_MoO_4_ (*Ln* = La, Ce, Pr, Nd) are given by Lysanova *et al.* (1983 ▶). The occurrence of a structural phase transition of LaBO_2_MoO_4_ was reported by Becker *et al.* (2008 ▶). For determinations of related structures, see: Palkina *et al.* (1979 ▶); Zhao *et al.* (2008 ▶, 2009 ▶) for LaBO_2_MoO_4_; Zhao *et al.* (2008 ▶) for CeBO_2_MoO_4_.

## Experimental

### 

#### Crystal data

NdMoBO_6_
*M* *_r_* = 346.99
Monoclinic, 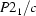

*a* = 10.1218 (19) Å
*b* = 4.1420 (5) Å
*c* = 11.896 (3) Å
β = 116.897 (14)°
*V* = 444.78 (16) Å^3^
*Z* = 4
Mo *K*α radiation
μ = 14.30 mm^−1^
*T* = 292 K
0.30 × 0.20 × 0.15 mm

#### Data collection

Enraf–Nonius CAD-4/MACH3 diffractometer
Absorption correction: ψ scan (*MolEN*; Fair, 1990 ▶) *T*_min_ = 0.487, *T*_max_ = 0.998
5004 measured reflections
1344 independent reflections
1268 reflections with *I* > 2σ(*I*)
*R*_int_ = 0.035
3 standard reflections every 100 reflections  intensity decay: 2.1%

#### Refinement

*R*[*F* ^2^ > 2σ(*F* ^2^)] = 0.025
*wR*(*F* ^2^) = 0.063
*S* = 1.22
1344 reflections
83 parameters
Δρ_max_ = 2.24 e Å^−3^
Δρ_min_ = −1.61 e Å^−3^

### 

Data collection: *MACH3* (Enraf–Nonius, 1993 ▶); cell refinement: *MACH3*; data reduction: *MolEN* (Fair, 1990 ▶); program(s) used to solve structure: *SHELXS97* (Sheldrick, 2008 ▶); program(s) used to refine structure: *SHELXL97* (Sheldrick, 2008 ▶); molecular graphics: *ATOMS* (Dowty, 2002 ▶); software used to prepare material for publication: *publCIF* (Westrip, 2010 ▶).

## Supplementary Material

Crystal structure: contains datablocks I, global. DOI: 10.1107/S1600536811017806/fi2107sup1.cif

Structure factors: contains datablocks I. DOI: 10.1107/S1600536811017806/fi2107Isup2.hkl

Additional supplementary materials:  crystallographic information; 3D view; checkCIF report

## Figures and Tables

**Fig. 1.**
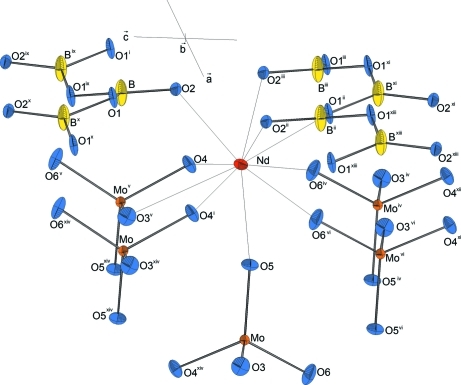
The structural units in NdBO2MoO4 with atomic numbering scheme (projection approximately along the b-axis). Atoms are drawn as 50% probability ellipsoids. Symmetry codes: (i) x, y + 1, z; (ii) -x, -y + 1, -z - 1; (iii) -x, -y, -z - 1; (iv) -x + 1, -y, -z - 1; (v) -x + 1, y - 1/2, -z - 1/2; (vi) -x + 1, -y + 1, -z - 1; (vii) -x + 1, y + 1/2, -z - 1/2; (viii) x, y - 1, z; (ix) -x, y - 1/2, -z - 1/2; (x) -x, y + 1/2, -z - 1/2; (xi) x, -y + 1/2, z + 1/2; (xii) x, -y - 1/2, z - 1/2; (xiii) x, 3/2 - y, z - 1/2; (xiv) 1 - x, 1/2 + y, z - 1/2.

**Fig. 2.**
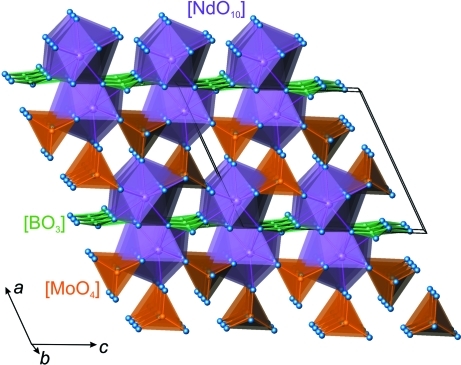
View of the crystal structure of NdBO2MoO4 along the b axis, showing chains of corner-sharing [BO3] groups (green), double-chains of face- and edge-sharing [NdO10] polyhedra (purple), and rows of isolated [MoO4] tetrahedra (orange), all of them running along the b axis. In a single row, [MoO4] tetrahedra are arranged with identical orientation, thus giving a polarity +b or -b to the row. Note the alternating polarity of the arrangement of [MoO4] tetrahedra rows along the c axis.

## supplementary crystallographic information

### Comment

In the family of lanthanide compounds *Ln*BO_2_MoO_4_ the crystal structure of LaBO_2_MoO_4_ was first described by Palkina *et al.* (1979), giving the polar space group *P*2_1_ and lattice constants *a* = 5.964 (1) Å, *b* = 4.147 (1) Å, *c* = 9.373 (3) Å, *β* = 99.28 (2)°. On the basis of X-ray powder diffraction data, Lysanova *et al.* (1983) claimed the isomorphism of the compounds *Ln*BO_2_MoO_4_ with *Ln* = La, Ce, Pr, Nd. The structure of LaBO_2_MoO_4_ was redetermined by Zhao *et al.* (2008), who reported a centrosymmetric structure with space group symmetry *P*2_1_/*c* with lattice constants *a* = 10.2968 (8) Å, *b* = 4.1636 (3) Å, *c* = 23.8587 (15) Å, *β* = 115.367 (3)°. These results were corroborated by Zhao *et al.* (2009), who, however, attributed this crystal structure to a high-temperature modification of LaBO_2_MoO_4_. The occurrence of a structural phase transition of LaBO_2_MoO_4_ was reported by Becker *et al.* (2008). Zhao *et al.* (2008) also presented the crystal structure of the related cerium compound, CeBO_2_MoO_4_, with space group *P*2_1_/*c* and lattice constants *a* = 10.2404 (15) Å. *b* = 4.1495 (4) Å, *c* = 11.9286 (14) Å, *β* = 116.100 (9)°, and thus showed, that the presumed isomorphism of the lanthanum and the cerium compound is not correct. However, the crystal structures are closely related, with an unit cell of LaBO_2_MoO_4_ which is doubled with respect to the unit cell of CeBO_2_MoO_4_. The result of the present study shows, that also NdBO_2_MoO_4_ is not isomorphic to LaBO_2_MoO_4_, but is isomorphic to CeBO_2_MoO_4_.

The crystal structure of NdBO_2_MoO_4_ consists of groups [BO_3_] and [MoO_4_] and tenfold coordinated neodymium atoms. The [BO_3_] groups are connected *via* common oxygen ligands to zigzag chains [B_2_O_4_]_∞_ along the *b* axis, with a periodicity of two [BO_3_] groups. The planar [BO_3_] groups are slightly distorted with O—B—O angles of 114.4 (4)°, 117.4 (4)° and 128.1 (4)°. They are linked by the common oxygen atom O1 with a bond angle B – O1 – B of 125.6 (3)° (see Fig. 1). The O1 ligands are connected to two boron atoms and one Nd atom, each, while the O2 ligands of the [BO_3_] groups are linked to three Nd atoms and one B, each. Mo atoms are tetrahedrally coordinated by the oxygen atoms O3, O4, O5 and O6 (see Fig. 1), with Mo—O distances ranging from 1.740 (4) Å (Mo—O3) to 1.816 (3) Å (Mo—O4) and angles O—Mo—O ranging from 96.3 (2)° (O4—Mo—O3) to 117.5 (2)° (O4—Mo—O6). The [MoO_4_] tetrahedra are arranged in rows that run parallel the *b* axis. Within a single row the [MoO_4_] tetrahedra are aligned with their Mo–O3 bonding directions pointing in the same direction approximately parallel to the *b* axis. Rows with opposite Mo–O3 bonding direction (*+b* and *-b*) alternate along the *c* axis, as shown in Fig. 2. Within a row the distance of a Mo atom to the O3 ligand of the neighbouring tetrahedron amounts 2.419 (3) Å. (Note that in Zhao *et al.* (2008) a distorted trigonal bipyramid is preferred as description of the coordination surrounding of Mo in CeBO_2_MoO_4_.) The oxygen atoms O3 and O5 serve as ligands of one Mo and one Nd atom, each, while the oxygen atoms O6 and O4 act as ligands for one Mo and two Nd atoms, each. The tenfold coordination of neodymium atoms in NdBO_2_MoO_4_ can be described as distorted bicapped square antiprism with Nd—O bonding distances ranging between 2.364 (3) Å (Nd—O2) and 2.981 (4) Å (Nd—O6). [NdO_10_] polyhedra are connected along the *b* axis by sharing three common oxygen ligands, namely O2, O6 and O4 (Fig. 1), with each neighbouring polyhedron, thus forming chains along the *b* axis. Two parallel chains are linked *via* edges (formed by two common O2 atoms) of the Nd coordination polyhedra, resulting in a double chain along *b*, see Fig. 2.

### Experimental

NdBO_2_MoO_4_ melts incongruently, therefore, single crystals of NdBO_2_MoO_4_ were grown from a melt with a composition differing from the crystal stoichiometry. A homogenized powder mixture of Nd_2_O_3_ (99.9%, Alfa Aesar), B_2_O_3_ (99.98%, Alfa Aesar) and MoO_3_ (99,95%, Alfa Aesar) in a molar ratio of 1: 1.375: 2.625 was heated in a covered platinum crucible in air atmosphere to 1423 K and subsequently cooled at a rate of 3 K h^-1^ to 1173 K. Transparent violet prismatic crystals of the title compound were separated mechanically from the surrounding flux. A suitable single-crystal was carefully selected using a polarizing microscope and mounted in a glass capillary.

### Refinement

The final difference maps indicate a maximum of 2.235 e Å^-3^ at a distance of 0.66 Å from Nd and a minimum of -1.611 e Å^-3^ at a distance of 1.06 Å of Nd.

### Crystal data

**Table:**

NdMoBO_6_	*F*(000) = 620
*M**_r_* = 346.99	*D*_x_ = 5.182 Mg m^−^^3^
Monoclinic, *P*2_1_/*c*	Mo *K*α radiation, λ = 0.71073 Å
Hall symbol: -P 2ybc	Cell parameters from 25 reflections
*a* = 10.1218 (19) Å	θ = 12.1–21.2°
*b* = 4.1420 (5) Å	µ = 14.30 mm^−^^1^
*c* = 11.896 (3) Å	*T* = 292 K
β = 116.897 (14)°	Prism, light violet
*V* = 444.78 (16) Å^3^	0.30 × 0.20 × 0.15 mm
*Z* = 4	

### Data collection

**Table:**

Enraf–Nonius CAD4/MACH3 diffractometer	1268 reflections with *I* > 2σ(*I*)
Radiation source: fine-focus sealed tube	*R*_int_ = 0.035
graphite	θ_max_ = 30.4°, θ_min_ = 2.3°
ω/2θ scans	*h* = −14→14
Absorption correction: ψ scan (*MolEN*; Fair, 1990)	*k* = −5→5
*T*_min_ = 0.487, *T*_max_ = 0.998	*l* = −16→16
5004 measured reflections	3 standard reflections every 100 reflections
1344 independent reflections	intensity decay: 2.1%

### Refinement

**Table:**

Refinement on *F*^2^	Primary atom site location: structure-invariant direct methods
Least-squares matrix: full	Secondary atom site location: difference Fourier map
*R*[*F*^2^ > 2σ(*F*^2^)] = 0.025	*w* = 1/[σ^2^(*F*_o_^2^) + (0.0304*P*)^2^ + 1.8082*P*] where *P* = (*F*_o_^2^ + 2*F*_c_^2^)/3
*wR*(*F*^2^) = 0.063	(Δ/σ)_max_ < 0.001
*S* = 1.22	Δρ_max_ = 2.24 e Å^−^^3^
1344 reflections	Δρ_min_ = −1.61 e Å^−^^3^
83 parameters	Extinction correction: *SHELXL97* (Sheldrick, 2008), Fc^*^=kFc[1+0.001xFc^2^λ^3^/sin(2θ)]^-1/4^
0 restraints	Extinction coefficient: 0.0428 (13)

### Special details

**Table:**

Geometry. All e.s.d.'s (except the e.s.d. in the dihedral angle between two l.s. planes) are estimated using the full covariance matrix. The cell e.s.d.'s are taken into account individually in the estimation of e.s.d.'s in distances, angles and torsion angles; correlations between e.s.d.'s in cell parameters are only used when they are defined by crystal symmetry. An approximate (isotropic) treatment of cell e.s.d.'s is used for estimating e.s.d.'s involving l.s. planes.
Refinement. Refinement of *F^2^* is done against ALL reflections. The weighted *R*-factor *wR* and goodness of fit *S* are based on *F^2^*, conventional *R*-factors *R* are based on *F*, with *F* set to zero for negative *F^2^*. The threshold expression of *F^2^ >2 σ(F^2^)* is used only for calculating *R*-factors(gt) *etc*. and is not relevant to the choice of reflections for refinement. *R*-factors based on *F^2^* are statistically about twice as large as those based on *F*, and *R*-factors based on ALL data will be even larger.

### Fractional atomic coordinates and isotropic or equivalent isotropic displacement parameters (Å^2^)

**Table:**

	*x*	*y*	*z*	*U*_iso_*/*U*_eq_	
Nd	0.19204 (2)	0.21132 (6)	−0.47183 (2)	0.00919 (11)	
Mo	0.64536 (4)	0.30466 (8)	−0.31742 (3)	0.00668 (12)	
B	−0.0028 (7)	0.3290 (12)	−0.3083 (5)	0.0182 (10)	
O1	−0.0326 (4)	0.6580 (8)	−0.3013 (3)	0.0120 (6)	
O2	−0.0035 (3)	0.2247 (7)	−0.4150 (3)	0.0099 (5)	
O3	0.6616 (4)	0.7223 (8)	−0.3016 (4)	0.0173 (7)	
O4	0.2605 (4)	−0.2856 (7)	−0.3501 (3)	0.0124 (6)	
O5	0.4556 (4)	0.2256 (9)	−0.3956 (3)	0.0156 (6)	
O6	0.7424 (4)	0.2194 (9)	−0.4084 (4)	0.0190 (7)	

### Atomic displacement parameters (Å^2^)

**Table:**

	*U*^11^	*U*^22^	*U*^33^	*U*^12^	*U*^13^	*U*^23^
Nd	0.00733 (14)	0.00900 (16)	0.00992 (14)	−0.00094 (6)	0.00274 (10)	−0.00205 (7)
Mo	0.00602 (17)	0.0087 (2)	0.00546 (17)	−0.00022 (10)	0.00270 (13)	−0.00094 (10)
B	0.040 (3)	0.007 (2)	0.013 (2)	0.0020 (19)	0.017 (2)	−0.0003 (17)
O1	0.0259 (16)	0.0056 (13)	0.0050 (12)	−0.0010 (11)	0.0074 (12)	−0.0004 (10)
O2	0.0108 (13)	0.0118 (14)	0.0084 (13)	−0.0018 (10)	0.0055 (11)	−0.0021 (10)
O3	0.0203 (16)	0.0125 (15)	0.0199 (16)	0.0003 (12)	0.0099 (14)	−0.0002 (12)
O4	0.0173 (15)	0.0091 (14)	0.0067 (13)	−0.0007 (10)	0.0018 (12)	−0.0017 (10)
O5	0.0066 (12)	0.0235 (17)	0.0137 (15)	−0.0009 (11)	0.0019 (11)	−0.0008 (12)
O6	0.0271 (18)	0.0192 (17)	0.0207 (17)	−0.0024 (13)	0.0197 (15)	−0.0039 (13)

### Geometric parameters (Å, °)

**Table:**

Nd—O2	2.364 (3)	B—O2	1.338 (6)
Nd—O5	2.399 (3)	B—O1^ix^	1.381 (6)
Nd—O4	2.431 (3)	B—O1	1.406 (6)
Nd—O4^i^	2.452 (3)	B—Nd^ii^	3.099 (6)
Nd—O1^ii^	2.498 (3)	B—Nd^iii^	3.315 (6)
Nd—O2^iii^	2.528 (3)	O1—B^x^	1.381 (6)
Nd—O6^iv^	2.551 (3)	O1—Nd^ii^	2.498 (3)
Nd—O3^v^	2.901 (4)	O2—Nd^iii^	2.528 (3)
Nd—O2^ii^	2.930 (3)	O2—Nd^ii^	2.930 (3)
Nd—O6^vi^	2.981 (4)	O3—Mo^i^	2.419 (3)
Mo—O3	1.740 (4)	O3—Nd^vii^	2.901 (4)
Mo—O5	1.745 (3)	O4—Mo^v^	1.816 (3)
Mo—O6	1.795 (3)	O4—Nd^viii^	2.452 (3)
Mo—O4^vii^	1.816 (3)	O6—Nd^iv^	2.551 (3)
Mo—O3^viii^	2.419 (3)	O6—Nd^vi^	2.981 (4)
Mo—Nd^vii^	3.5017 (8)		
			
O2—Nd—O5	145.40 (12)	O4^vii^—Mo—Nd^vi^	128.02 (11)
O2—Nd—O4	84.21 (11)	O3^viii^—Mo—Nd^vi^	120.27 (9)
O5—Nd—O4	80.04 (12)	Nd^vii^—Mo—Nd^vi^	103.052 (17)
O2—Nd—O4^i^	81.98 (11)	Nd^v^—Mo—Nd^vi^	135.816 (15)
O5—Nd—O4^i^	77.71 (12)	O3—Mo—Nd	99.98 (12)
O4—Nd—O4^i^	116.06 (13)	O5—Mo—Nd	7.61 (12)
O2—Nd—O1^ii^	95.08 (11)	O6—Mo—Nd	121.06 (13)
O5—Nd—O1^ii^	117.89 (12)	O4^vii^—Mo—Nd	113.32 (11)
O4—Nd—O1^ii^	134.27 (11)	O3^viii^—Mo—Nd	87.93 (9)
O4^i^—Nd—O1^ii^	109.02 (10)	Nd^vii^—Mo—Nd	114.650 (15)
O2—Nd—O2^iii^	68.97 (12)	Nd^v^—Mo—Nd	105.813 (15)
O5—Nd—O2^iii^	131.40 (11)	Nd^vi^—Mo—Nd	116.653 (16)
O4—Nd—O2^iii^	69.84 (11)	O3—Mo—Nd^iv^	122.17 (12)
O4^i^—Nd—O2^iii^	149.91 (11)	O5—Mo—Nd^iv^	102.87 (12)
O1^ii^—Nd—O2^iii^	67.44 (11)	O6—Mo—Nd^iv^	20.25 (12)
O2—Nd—O6^iv^	129.71 (11)	O4^vii^—Mo—Nd^iv^	113.64 (11)
O5—Nd—O6^iv^	72.70 (12)	O3^viii^—Mo—Nd^iv^	60.20 (9)
O4—Nd—O6^iv^	70.40 (12)	Nd^vii^—Mo—Nd^iv^	134.533 (16)
O4^i^—Nd—O6^iv^	148.09 (12)	Nd^v^—Mo—Nd^iv^	94.773 (16)
O1^ii^—Nd—O6^iv^	75.67 (12)	Nd^vi^—Mo—Nd^iv^	60.259 (12)
O2^iii^—Nd—O6^iv^	61.79 (10)	Nd—Mo—Nd^iv^	110.359 (15)
O2—Nd—O3^v^	75.40 (11)	O2—B—O1^ix^	128.1 (4)
O5—Nd—O3^v^	70.07 (11)	O2—B—O1	117.4 (4)
O4—Nd—O3^v^	58.78 (10)	O1^ix^—B—Nd^ii^	155.4 (4)
O4^i^—Nd—O3^v^	57.31 (10)	O1^ix^—B—Nd^iii^	102.0 (3)
O1^ii^—Nd—O3^v^	163.84 (10)	O1—B—Nd^iii^	129.6 (4)
O2^iii^—Nd—O3^v^	119.20 (10)	Nd^ii^—B—Nd^iii^	80.37 (14)
O6^iv^—Nd—O3^v^	120.47 (11)	O1^ix^—B—Nd	120.0 (3)
O2—Nd—O2^ii^	69.96 (11)	O1—B—Nd	111.8 (3)
O5—Nd—O2^ii^	122.40 (10)	Nd^ii^—B—Nd	84.34 (13)
O4—Nd—O2^ii^	154.13 (10)	Nd^iii^—B—Nd	74.17 (11)
O4^i^—Nd—O2^ii^	62.95 (10)	O2—B—Nd^xi^	144.1 (4)
O1^ii^—Nd—O2^ii^	50.40 (10)	O1—B—Nd^xi^	90.2 (3)
O2^iii^—Nd—O2^ii^	98.46 (10)	Nd^ii^—B—Nd^xi^	142.25 (16)
O6^iv^—Nd—O2^ii^	125.50 (11)	Nd^iii^—B—Nd^xi^	132.90 (16)
O3^v^—Nd—O2^ii^	113.50 (9)	Nd—B—Nd^xi^	117.93 (18)
O2—Nd—O6^vi^	120.96 (10)	B^x^—O1—B	125.6 (3)
O5—Nd—O6^vi^	73.22 (11)	B^x^—O1—Nd^ii^	132.3 (3)
O4—Nd—O6^vi^	152.84 (11)	B—O1—Nd^ii^	101.4 (3)
O4^i^—Nd—O6^vi^	62.98 (10)	B^x^—O1—Nd^xi^	57.4 (2)
O1^ii^—Nd—O6^vi^	59.13 (11)	B—O1—Nd^xi^	68.4 (3)
O2^iii^—Nd—O6^vi^	126.02 (10)	Nd^ii^—O1—Nd^xi^	169.04 (12)
O6^iv^—Nd—O6^vi^	96.66 (11)	B^x^—O1—Nd	137.1 (3)
O3^v^—Nd—O6^vi^	114.35 (10)	B—O1—Nd	49.7 (3)
O2^ii^—Nd—O6^vi^	52.35 (9)	Nd^ii^—O1—Nd	78.20 (8)
O3—Mo—O5	105.76 (17)	Nd^xi^—O1—Nd	96.89 (8)
O3—Mo—O6	102.12 (17)	B—O2—Nd	129.3 (3)
O5—Mo—O6	114.36 (17)	B—O2—Nd^iii^	114.5 (3)
O3—Mo—O4^vii^	96.26 (16)	Nd—O2—Nd^iii^	111.03 (12)
O5—Mo—O4^vii^	116.79 (16)	B—O2—Nd^ii^	84.3 (3)
O6—Mo—O4^vii^	117.52 (17)	Nd—O2—Nd^ii^	110.04 (11)
O3—Mo—O3^viii^	169.5 (2)	Nd^iii^—O2—Nd^ii^	98.46 (10)
O5—Mo—O3^viii^	82.75 (15)	Mo—O3—Mo^i^	169.5 (2)
O6—Mo—O3^viii^	79.30 (15)	Mo—O3—Nd^vii^	94.65 (15)
O4^vii^—Mo—O3^viii^	74.10 (13)	Mo^i^—O3—Nd^vii^	94.88 (12)
O3—Mo—Nd^vii^	55.67 (13)	Mo—O3—Nd^vi^	92.60 (14)
O5—Mo—Nd^vii^	121.91 (12)	Mo^i^—O3—Nd^vi^	84.42 (10)
O6—Mo—Nd^vii^	123.02 (13)	Nd^vii^—O3—Nd^vi^	131.41 (12)
O4^vii^—Mo—Nd^vii^	40.63 (10)	Mo^v^—O4—Nd	110.25 (14)
O3^viii^—Mo—Nd^vii^	114.71 (9)	Mo^v^—O4—Nd^viii^	133.69 (16)
O3—Mo—Nd^v^	123.03 (13)	Nd—O4—Nd^viii^	116.06 (13)
O5—Mo—Nd^v^	105.84 (12)	Mo^v^—O4—Nd^iii^	117.45 (14)
O6—Mo—Nd^v^	106.15 (13)	Nd—O4—Nd^iii^	71.19 (8)
O4^vii^—Mo—Nd^v^	26.80 (10)	Nd^viii^—O4—Nd^iii^	78.89 (8)
O3^viii^—Mo—Nd^v^	47.32 (9)	Mo—O5—Nd	166.9 (2)
Nd^vii^—Mo—Nd^v^	67.430 (16)	Mo—O6—Nd^iv^	145.65 (19)
O3—Mo—Nd^vi^	62.27 (12)	Mo—O6—Nd^vi^	115.69 (16)
O5—Mo—Nd^vi^	114.60 (12)	Nd^iv^—O6—Nd^vi^	96.66 (11)
O6—Mo—Nd^vi^	41.02 (12)		

Symmetry codes: (i) *x*, *y*+1, *z*; (ii) −*x*, −*y*+1, −*z*−1; (iii) −*x*, −*y*, −*z*−1; (iv) −*x*+1, −*y*, −*z*−1; (v) −*x*+1, *y*−1/2, −*z*−1/2; (vi) −*x*+1, −*y*+1, −*z*−1; (vii) −*x*+1, *y*+1/2, −*z*−1/2; (viii) *x*, *y*−1, *z*; (ix) −*x*, *y*−1/2, −*z*−1/2; (x) −*x*, *y*+1/2, −*z*−1/2; (xi) *x*, −*y*+1/2, *z*+1/2.
